# Physical properties, engine performance, and exhaust emissions of waste fish oil biodiesel/bioethanol/diesel fuel blends

**DOI:** 10.1038/s41598-023-41280-5

**Published:** 2023-08-28

**Authors:** Davood Tarangan, Mohammad Amin Sobati, Shahin Shahnazari, Barat Ghobadian

**Affiliations:** 1 School of Chemical Engineering, Iran University of Science and Technology (IUST), Tehran, Iran; 2 Faculty of Agricultural Engineering, Tarbiat Modares University, Tehran, Iran

**Keywords:** Chemical engineering, Mechanical engineering, Environmental impact, Physical chemistry

## Abstract

In the current study, the physicochemical, engine performance, and exhaust emission of different ternary fuel blends containing waste fish oil (WFO) biodiesel, bioethanol, and petro-diesel have been investigated. WFO Biodiesel was prepared from waste fish oil via transesterification method. Different physiochemical properties including the kinematic viscosity, density, flash point, pour point, cloud point, and heat value have been measured for different fuel blends and compared with the neat petro-diesel. The performance and exhaust emission of engine have been also studied using different fuel blends using a single-cylinder diesel engine in full load condition at 1800 rpm. It was found that the engine torque, engine power, and thermal efficiency of the ternary fuel blends was reduced by 2.45%, 9.25%, 2.35% averagely in comparison with the neat petro-diesel, respectively. The average break specific fuel consumption was also increased by 10.44% compared to the neat petro-diesel. The emission of carbon monoxide (CO), carbon dioxide (CO_2_), unburned hydrocarbons (UHC), and nitrogen oxides (NO_x_) was also measured. It was also found that the utilization of ternary fuel blends results in a considerable reduction in CO and UHC emission by 50.55% and 43.87% on average compared to the neat petro-diesel, respectively. The emission of NO_x_ was also increased by 28.25% on average compared to the neat petro-diesel. It was also found that the NO_x_ emission can be adjusted by tuning the WFO biodiesel and bioethanol contents of the ternary fuel blends.

## Introduction

Depleting the fossil fuel resources, the pollution caused by the fossil fuels combustion such as carbon oxides (COx), nitrogen oxides (NO_x_), unburned hydrocarbons (UHC), particular matter (PM) emissions are the most concerning issues about the fossil fuels utilization^1,2^. Therefore, the researchers are always looking for appropriate alternatives for the fossil fuels. Solar energy, wind energy, waves energy, and geothermal energy are interesting renewable energy sources. The biofuels as one of the renewable energy resources, can be applied in similar fossil fuel applications because the biofuel specifications can be similar to the diesel fuel. Depending on the type of biofuel and its application, the biofuels can be used as single or in combination with fossil fuels. For example, the neat biodiesel can be applied in a boiler combustion system but the mixture of biodiesel and petrodiesel can be applied in an internal combustion engine^3–6^.

Bioethanol is renewable and ecofriendly biofuel which can be produced from sugar, starch, and cellulosic sources by fermentation and hydrolysis processes^6^. Various raw materials such as sugar, corn, wheat, potato, stem, hay, agricultural wastes, molasses, macroalgae, microalgae, and seaweed are potential feedstocks for the bioethanol production^7,8^. The feedstocks like sugar, corn, and potato are edible resources that fall into the first generation feedstocks for the biofuel production, which are not recommended for the bioethanol production due to food versus fuel debate. The application of agricultural wastes, molasses, macroalgae, microalgae, and seaweed as biofuel feedstocks could be a proper solution for this problem^9^. Bioethanol cannot be used as a single fuel in the diesel engines and it should be blended with the petro-diesel^1,10^. Using diesel/bioethanol blended fuel have some advantages such as increase in the rate of premixed combustion, improvement of the thermal efficiency and also reducing the smoke exhaust^1^. However, there are some challenges in the bioethanol addition to the diesel fuel such as limited solubility of bioethanol in the diesel, possible fuel phase separation in cold conditions, and negative effect of bioethanol on some fuel specifications such as Cetane number, heat value, and flash point^11,12^. Addition of emulsifiers or co-solvents is recommended to solve the phase separation issue in the diesel/bioethanol fuel blend. Among different potential co-solvents, the application of esters is recommended because esters can improve the blend properties and covering the negative effects of bioethanol on the fuel blend^10,13^.

Biodiesel is another type of biofuel that mainly consists of triglyceride methyl esters, which is produced from the plant oils or animal fats by different processes such as micro-emulsion, thermal cracking, and transesterification. Waste fish oil (WFO) is produced as a byproduct in large quantities in the fish processing industries. This byproduct is more valuable compared to other byproducts such as fish silage or fish fertilizer^14^. It should be noted that WFO is not a good feed for the pharmaceutical and functional food industries due to its low content of Eicosapentaenoic acid (EPA) and docosahexaenoic acid (DHA) and subsequent low omega-3 concentration. WFO is categorized in the second and third generations feedstocks for the biodiesel production including non-edible resources in which the food versus fuel dilemma is not encountered^15^. The application of WFO in the biodiesel production can reduce the costs of biodiesel production noticeably^14^. Biodiesel is usually used in combination with petro-diesel fuel in the diesel engine. The application of biodiesel in the engines leads to an increase in NO_x_ emissions^8,16,17^. According to the literature, the application of the diesel/bioethanol/biodiesel fuel blend has better properties and performance compared to the diesel/biodiesel or the diesel/bioethanol fuel blends^18^.

Hulwan et al.^12^ studied the performance, emissions, and combustion characteristics of Jatropha biodiesel/ethanol/diesel fuel blend in a multi cylinder DI diesel engine. According to their results, the presence of biodiesel in the fuel blends results in an increase in the ethanol solubility in the fuel blend. BSFC of fuel blends increased by an increment in the ethanol content of the fuel blend. It was also found that the BTE increases at high load for all fuel blends compared to the neat diesel. Smoke was also decreased noticeably for all fuel blends compared to the neat diesel. According to their results, the CO emission for the fuel blends was increased significantly at low load and decreased slightly at high load compared to the neat diesel^12^. Kwanchareon et al.^19^ investigated the emission characteristics of palm oil biodiesel/ethanol/diesel in the diesel engine. According to this study, the biodiesel acts as an effective additive for the stabilization of ethanol in the fuel blend. According to the results, CO and HC emissions of fuel blends reduced significantly at high engine loads compared to the petro-diesel. It was also found that the NO_x_ emission of the fuel blends increases compared to the neat diesel^19^. Aydin et al.^6^ have checked the emission and performance of an engine fueled with safflower biodiesel/ethanol/diesel. According to their results, no significant variation was observed in the engine torque and power using the fuel blends compared to the neat diesel. Regarding to the specific fuel consumption (SFC), an increase in the SFC was observed for the fuel blends compared to the neat diesel. CO_2_ and HC emissions of the fuel blends are generally more than the neat diesel. SO_2_ emission of the fuel blends is lower compared to the neat diesel^6^. Guarieiro et al.^10^ studied the emissions of a diesel engine fueled by ternary blends of (soybean biodiesel or castor biodiesel or residual biodiesel or soybean oil or castor oil)/ethanol/diesel. Based on their results, the utilization of diesel/ethanol blend fuel leads to the highest reduction in the NO_x_ emission in comparison with the neat diesel. Regarding the CO_2_ emission, the results show that the fuel blends emission was decreased in the range of 5–24% and 4–6% at 1800 rpm and 2000 rpm compared to the neat diesel, respectively. No sensible difference between the CO emission of the blend fuels and the neat diesel was observed. More carbonyl compounds emission was observed for all examined blend fuel samples compared to the neat diesel^10^. Subbaiah et al.^2^ studied the emission and performance of an engine using rice bran oil biodiesel/ethanol/diesel fuel blends. Based to their report, BTE and BSFC of all examined fuel blends are higher compared to the neat diesel. It was also found that the NO_x_ emission of all fuel blends were lower compared to the neat diesel at low loads and higher compared to the neat diesel at high loads. CO_2_ emission of all fuel blends is higher compared to the neat diesel. CO emission of all fuel blends is lower compared to the neat diesel. The HC emission of all fuel blends are higher compared to the neat diesel at low loads and lower compared to the neat diesel at high loads^2^. Recently, Sathiyaseelan et al.^20^ studied the performance, emission, and combustion characteristics of diesel/WFO biodiesel/ethanol fuel blends in DI diesel engine at various compression ratio. According to their report, the application of neat diesel result in the highest peak in the cylinder pressure and highest heat release rate (HRR) considering all examined fuel samples except for D91.25B7.5E1.25 at compression ratio of 18. Diesel fuel has the highest BTE and the lowest BSFC compared to the other examined fuel samples. Moreover, the lowest CO, and NO_x_ emission was observed for D86.25B12.5E1.25 fuel blend considering all examined fuel samples. Table 1 shows a brief summary of the previous studies on the performance and emission of biodiesel/bioethanol/diesel fuel blends.

**Table 1:** Previous studies on the ternary fuel blends of petro-diesel/bioethanol/biodiesel.

Researchers	Fuel formulation	Investigated engine performance parameter	Investigated engine emissions
Hulwan et al.^12^	Diesel/ethanol/biodiesel blends (D70/E20/B10, D50/E30/B20, D50/E40/B10)	BSFC, BTE	Smoke, NO_x_, CO, CO_2_
Kwanchareon et al.^19^	Diesel/ethanol/biodiesel blends (D90/B10, D90/B5/E5, D90/E10, D85/B15, D85/B10/E5, D85/B5/E10, D850/E15, D80/B15/E5, D80/B10/E10, D80/B5/E15)	–	NO_x_, CO, HC
Aydin et al.^11^	Diesel/safflower biodiesel/ethanol blends (B_2.5_ M_2.5_ D_95_, B_5_ M_5_ D_90_, B_5_ M_2.5_ D_92.5_, B_2.5_ M_5_ D_92.5_)	Torque, Power, SFC	NO_x_, CO, CO_2_, HC, SO_2_, O_2_
Guarieiro et al.^10^	Diesel/ethanol—90/10%, Diesel/ethanol/soybean biodiesel—80/15/5%, diesel/ethanol/castor biodiesel—80/15/5%, diesel/ethanol/residual biodiesel—80/15/5%, diesel/ethanol/soybean oil—90/7/3%, and diesel/ethanol/castor oil—90/7/3%	–	18 Carbonyl compounds, NO_x_, CO, CO_2_
Subbaiah et al.^2^	Diesel/rice bran oil biodiesel/ethanol blends B10E5, B10E10, B10E15	BTE, BSFC	HC, NO_x_, CO, CO_2_
Sathiyaseelan et al.^20^	D91.25B7.5E1.25, D87.5B7.5E5, D86.25B12.5E1.25, D82.5B12.5E5, D81.25B17.5E1.25, D77.5B17.5E5	BTE, BSFC	CO, UHC, CO_2_, NO_x_
Paul et al.^21^	D100, D45E5B50, D40E10B50, D35E15B50, D30E20B50	BTE, BSEC	HC, CO, NO_x_
Yilmaz et al.^22^	Diesel, BDE3, BDE5, BDE15, BDE25		HC, CO, NO
Paul et al.^23^	Diesel, D95E5, D90E10, D45E15B40, D30E20B50	BTE, BSFC	HC, CO_2_, NO_x_, smoke
Krishna et al.^24^	Diesel, BDE_opt_, BDE_6_, BDE_7_, BDE_8_,BDE_9_	SFC, TE	NO_x_,CO,CO_2_
Ağbulut et al.^25^	D100, D80C20, D90E10, D70C20E10	Torque, Power	O_2_,CO, HC, NO_x_

In summary, many researchers have focused on different diesel/biodiesel fuel blends, however, it was found that the addition of bioethanol to the biodiesel/diesel can improve the fuel blends performance such as reduction in NO_x_ emission. Therefore, more researches should be carried out on the ternary fuel blend (i.e., biodiesel/bioethanol/diesel) as an appropriate alternative for the petro-diesel.

Based on our literature survey, the application WFO biodiesel in diesel/biodiesel/bioethanol fuel blends is only limited Sathiyaseelan et al.^20^ study which recently examined this ternary fuel blends in limited concentration ranges of WFO biodiesel and ethanol in the blend. Therefore, the available data on the physical property specifications, performance, and emission of WFO biodiesel/bioethanol/diesel are limited. In this regard, the present study is conducted to fill this research gap by considering ternary fuel blends of bioethanol, WFO biodiesel, and petro-diesel in more wide concentration ranges. In this regard, the physical property specifications (i.e., density, kinematic viscosity, pour point, cloud point, closed cup flash point, and heat value), the engine performance (torque, power, BSFC, and BTE), exhaust emission (CO, CO_2_, UHC, and NO_x_) were investigated for different WFO biodiesel/bioethanol/diesel fuel samples.

## Experimental

### Material

Neat petro-diesel was supplied by Tehran refining company (Tehran, Iran). Waste Fish Oil (WFO), for biodiesel production, was supplied from Daneh Talayi Chabahar Company (Chabahar, Iran). Methanol (99.8%), for the esterification and the transesterification reactions, and 2-propanol (99.8%), for determination of the free fatty acids content in the WFO, were purchased from Dr. Mojallalli Industrial Chemical complex company (Iran). Bioethanol (99.8%) was supplied from Kimia Alcohol Zanjan Company (Iran). Sulfuric acid (99.6%), as the catalyst in the esterification reactions, and potassium hydroxide (KOH), as the catalyst in the transesterification reactions, were purchased from Merck Company (Germany).

### Preparation of waste fish oil (WFO) biodiesel

WFO which is a byproduct of the fish powder production company. WFO was heated in the production stages in the plant and was passed through a micro filter in order to recover the fish powders from the existing in the oil. Therefore, the purchased WFO is free from the solid impurities and water.

In this study, WFO biodiesel was produced by transesterification method. Free fatty acids (FFA) content of WFO is high, which results in undesirable reaction in the transesterification stage. Therefore, the FFA content of WFO should be decreased to < 1% to maximize the efficiency of biodiesel production via transesterification^10^. FFA content of WFO is determined by a simple acid/base neutralization experiment. FFA is determined by Eq. (1):

1 $$ \% FFA = \frac{0.5 \times A \times N \times Wcat}{W}. $$

In which, A is the required volume of the solution for the titration of WFO in mL, W is the amount of WFO sample in g, N is the concentration of the titration solution in normality scale, and W_cat_ is the molecular weight of the catalyst. The titration solution is 0.1 N. Potassium hydroxide (KOH) and 2-propanol alcohol were used as the catalyst and solvent in the titration, respectively.

Since the FFA content of WFO is higher than 1%, the esterification processing step is required in one or more stages to reduce the FFA content of WFO < 1%. The esterification of WFO was carried out in a 70 L batch reactor equipped with a mechanical stirrer with 300 rpm stirring rate and a recycle stream. In the esterification reaction, the methanol to WFO ratio was 9 to 1 in the presence of 1 wt% KOH/sulfuric acid solution at 55 °C for 1 h^4,14,26^. After termination of the esterification reaction, the unreacted methanol and produced water should be separated from the esterified WFO. Therefore, the reactor content was introduced into a decanter for the phase separation. The treated WFO phase was separated after 24 h. After WFO esterification, the FFA content of treated WFO was reduced to 0.96% which is lower than 1%. Therefore, the treated WFO is ready for transesterification. The transesterification of treated WFO was carried out considering the methanol to WFO ratio of 6:1 in the presence of 1 wt% KOH solution at 60 °C for 1 h^4,14,26^. The products of transesterification reaction are biodiesel and glycerin. The glycerin phase was separated from the WFO biodiesel after 24 h. The final step in the WFO biodiesel preparation was water washing in order to remove the remaining catalyst, alcohol, soap, and glycerin from the WFO biodiesel. In this regard, the WFO biodiesel was mixed with water (water to biodiesel ratio of 2:1) at 60 °C for 1.5 h. The washing water was separated from the WFO biodiesel by decantation. The treated WFO biodiesel was heated at 85 °C for 8 h for separation of the remaining water from the WFO biodiesel. The final WFO biodiesel was used in the fuel blends.

### Engine test and emission measurement system

An Air-cooled single-cylinder diesel engine (model 3LD510 from Lombardini Company) equipped with a dynamometer (model WE400 from Mobtakeran Pars Andish Company) was employed in order to determine the rotational speed, the torque, and power of the diesel engine using different fuel blends. A specific software was employed for other engine performance parameter calculations including brake power (BP), specific fuel consumption (SFC), brake specific fuel consumption (BSFC), and brake thermal efficiency (BTE) using the following equations^4^. The main specifications of the employed diesel engine and dynamometer can be found in the supporting information file.

2 $$ BP = \frac{2\pi TN}{{60,000}} $$

3 $$ SFC = \frac{{M_{f} }}{P} $$

4 $$ BSFC = \frac{{M_{f} }}{BP} $$

5 $$ BTE = \frac{3600}{{H_{v} \times BSFC}} $$

MAHA-MGT5 device was used to measure the extent of engine emission using different fuel blends. MAHA-MGT5 is capable to determine the CO_2_, CO, and HC emission by infrared technology as well as O_2_ and NO_x_ using electrochemical sensors. The detailed specifications of the applied emission sensors including the detection range and accuracy can be found in the supporting information file.

**Figure 1:**
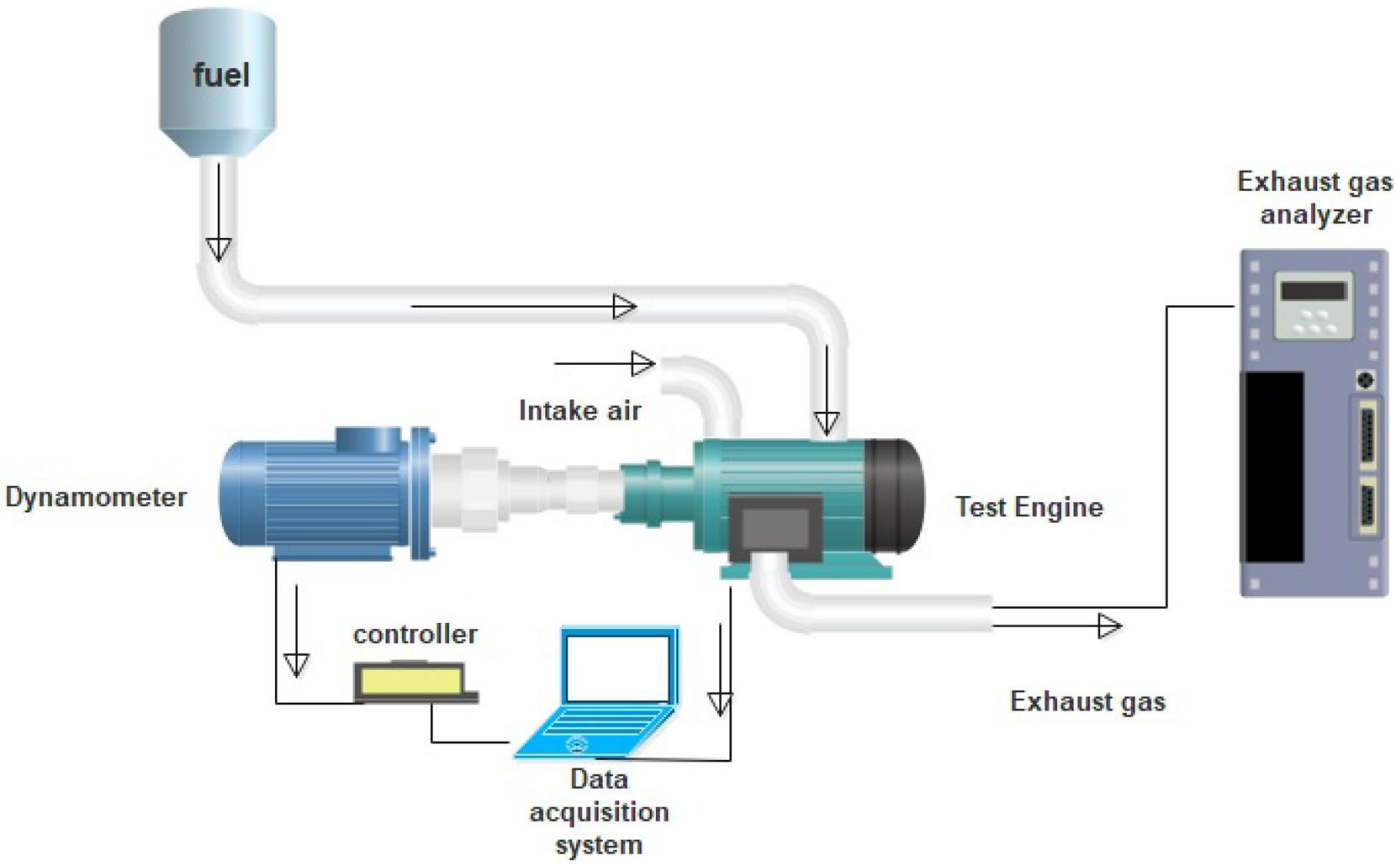
The experimental setup employed for the engine tests.

Figure 1 illustrates the single-cylinder diesel engine setup applied for evaluation of the engine performance and emission using different fuel blends.

It should be noted that in each engine test, the engine was held unloaded for 15 min until the oil temperature reaches 70 °C. This condition is so-called warming up the engine. Then, the full load condition was imposed on the engine and the engine speed was set to 1800 rpm. Afterwards, the engine performance and emission were measured at steady state condition. It should be added that the reliability of the experimental measurements was confirmed by repeating the experimental runs for the fuel blends. The relative standard deviation was in the acceptable range of 1–4%. It should be mentioned that the engine tests were carried out in the Renewable Energy laboratory, Bioenergy Research Center, Tarbiat Modares University, Tehran, Iran.

### Fuel composition and physico-chemical properties measurement

The fatty acid profile of the WFO biodiesel can affect the engine emission and performance characteristics^3,8^. In the present study, Clarus 580 gas chromatography (GC) manufactured by Perkin Elmer Company was used to evaluate the fatty acid profile of WFO biodiesel. The GC was equipped with a flame ionization detector (FID) to specify the WFO different biodiesel compounds. A special GC column (model CP 9080 from Varian Company with 30 m length, internal diameter of 0.32 mm, and static phase thickness of 0.25 μm) was employed. Helium was used as the carrier gas. The temperature program of the column was adjusted according to EN 14,103 standard.

Important physicochemical properties of fuels are density, kinematic viscosity, pour point, cloud point, flash point, and heat value^3,4,27^. The density of different fuel blends was determined using SVM-3000 Stabinger viscometer (Anton paar Company) based on ASTM D4052 with precision of 0.0001 g/cm^3^. The kinematic viscosity of different fuel blends was determined using SVM-3000 Stabinger viscometer (Anton paar Company) based on ASTM D 455-06 with an accuracy of 0.01 cst. The pour point of fuel blends was determined according to ASTM D97. The measurement of the cloud point of fuel samples was carried out based on ASTM D2500 standard. The flash point of fuel blends was measured based on closed-cup method according to ASTM D93 using SKY1002-I flash point apparatus from Shanghai shenakai with an accuracy of ± 2 °C. The calorific value of the fuel blends was measured using Gallenkamp calorimeter bomb apparatus with an accuracy of ± 0.1%.

## Results and discussion

### Fuels properties

According to the GC analysis, the main fatty acid esters of WFO biodiesel are palmitic acid, and trans-9-Elaidic acid methyl ester. The presence of Myristic acid methyl ester in the WFO biodiesel is also confirmed. More details regarding the fatty acid profiles and the compositional analysis of the WFO biodiesel can be found in the supporting information file.

**Figure 2:**
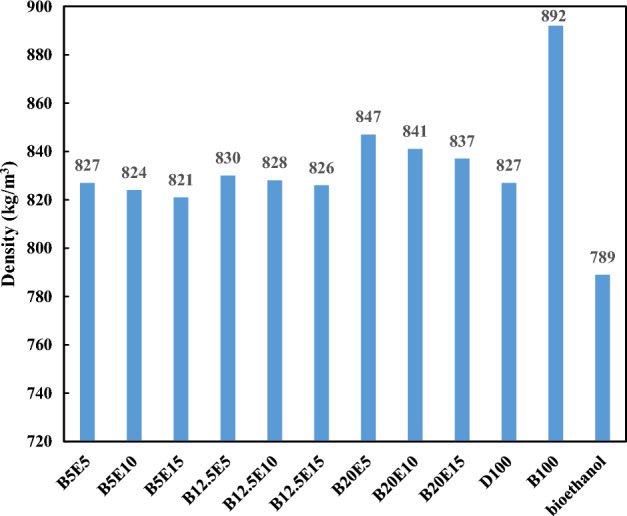
The density of different ternary fuel blends.

**Figure 3:**
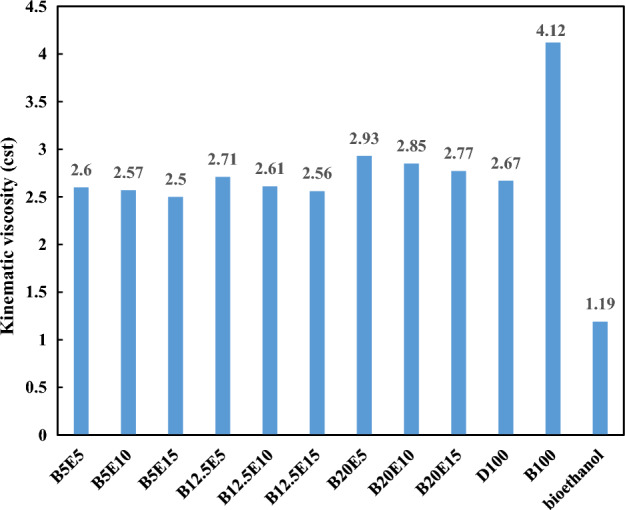
Kinematic viscosity of different ternary fuel blends.

**Figure 4:**
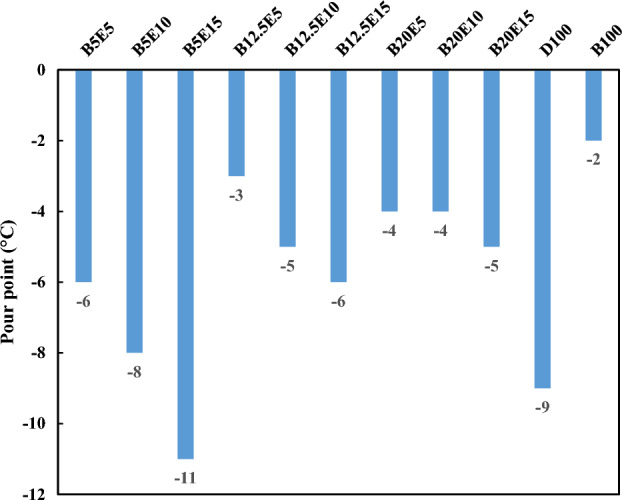
Pour point of different ternary fuel blends.

**Figure 5:**
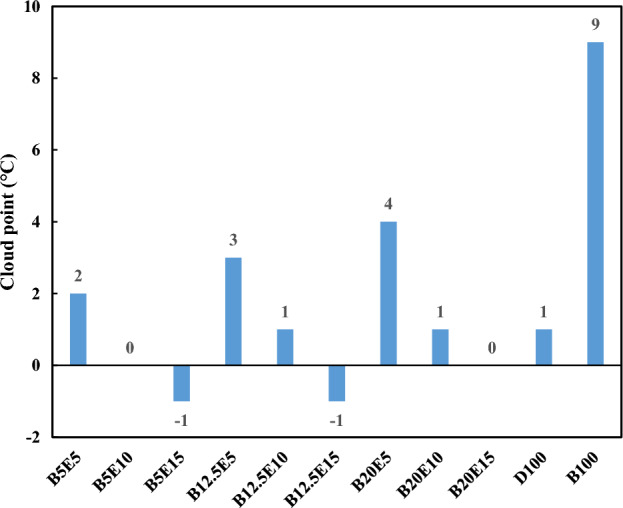
Cloud point of different ternary fuel blends.

**Figure 6:**
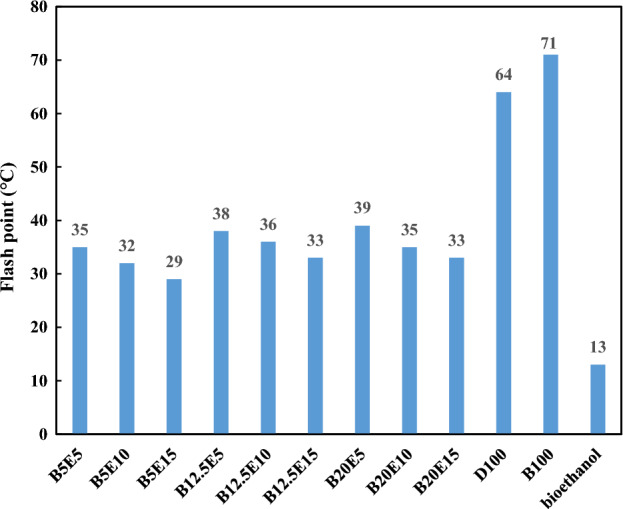
Closed cup flash point of different ternary fuel blends.

**Figure 7:**
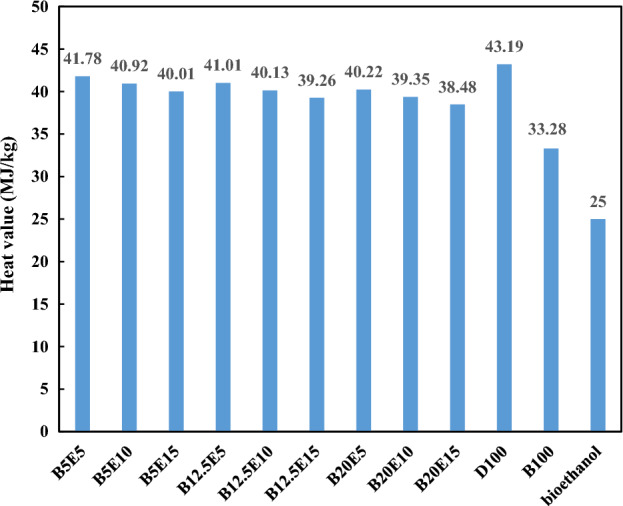
Heat value of different ternary fuel blends.

In the present study, different fuel blends were prepared by mixing different concentration of WFO biodiesel, bioethanol, and petro-diesel. Table 2 shows the detailed composition of different WFO biodiesel/bioethanol/diesel fuel samples. Afterwards, different specifications such as density, kinematic viscosity, pour point, cloud point, and flash point were measured for different fuel blends according to ASTM D 4052, ASTM D 445-06, ASTM D 97, ASTM D 2500, and ASTM D93 standards, respectively. The results of these measurements are presented in Figs. 2, 3, 4, 5, 6 and 7 for different examined fuel samples. The range of WFO biodiesel in the ternary fuel blends is 5–20%. The range of bioethanol in the ternary fuel samples is 5–15%. These selected concentration ranges are considerably wider compared to Sathiyaseelan et al.^20^ study.

**Table 2:** Different examined fuel samples.

Fuel samples	Composition
D100	100% vol. diesel
B100	100% vol. WFO biodiesel
Bioethanol	100% volume bioethanol
B5E5	90% vol. diesel + 5% vol. biodiesel + 5% vol. bioethanol
B5E10	85% vol. diesel + 5% vol. biodiesel + 10% vol. bioethanol
B5E15	80% vol. diesel + 5% vol. biodiesel + 15% vol. bioethanol
B12.5E5	82.5% vol. diesel + 12.5% vol. biodiesel + 5% vol. bioethanol
B12.5E10	77.5% vol. diesel + 12.5% vol. biodiesel + 10% vol. bioethanol
B12.5E15	72.5% vol. diesel + 12.5% vol. biodiesel + 15% vol. bioethanol
B20E5	75% vol. diesel + 20% vol. biodiesel + 5% vol. bioethanol
B20E10	70% vol. diesel + 20% vol. biodiesel + 10% vol. bioethanol
B20E15	65% vol. diesel + 20% vol. biodiesel + 15% vol. bioethanol

Figure 2 shows the density of different examined fuel samples. It should be noted that the fuel density directly affect the injection timing, and spray quality of fuels in the combustion chamber^28,29^. Fuel density also affect the exhaust emissions. Using high density fuel results in increasing the amount of fuel entering the combustion chamber and it gets the oxygen to fuel ratio out of balance, which leads to incomplete combustion and subsequent increment in the emission of unburned hydrocarbons^28^. According to Fig. 2, the density of fuel blends increases by an increment in the WFO biodiesel content of blends because the density of WFO biodiesel is higher than the neat diesel due to higher molecular weight of WFO biodiesel compared to neat diesel^30^. The density of biodiesel produced from different sources are in the same range but the WFO biodiesel (density = 892 kg/m^3^) is more suitable for the fuel blends preparation compared to the soybean biodiesel (density = 913 kg/m^3^) and the pongamia biodiesel (density = 931 kg/m^3^)^8^. On the other hand, the density of ternary fuel blends decreases by an increment in the bioethanol content of blend due to lower density of bioethanol compared to diesel and WFO biodiesel. The addition of bioethanol leads to an improvement in the fuel blend/air mixing due to its low density which results in more complete combustion close to the stoichiometric ratio and subsequent decrease in the exhaust smoke^31^. According to Fig. 2, it was also found that the bioethanol addition to the ternary fuel blends neutralizes the density increment of WFO biodiesel addition. In this regard, the lowest value of density is observed for B5E15 fuel blends.

Figure 3 shows the kinematic viscosity of different fuel samples. The fuel viscosity is an important parameter which affects the spray quality and consequently the fuel and air mixing^14^. The viscosity of WFO biodiesel is higher compared to petro-diesel, which may leads to incomplete combustion by reducing the fuel and air mixing quality in the combustion chamber and increment in the exhaust emission^3,32^. Heat release rate (HRR) and cylinder pressure are the main combustion characteristics which are influenced by the fuel viscosity. Increasing the biodiesel content of the fuel blends leads to poor atomization of the fuel sample due to its high viscosity and results in an increment in the ignition delay and subsequent lower HRR and lower cylinder pressure^33,34^. On the other hand, the oxygen concentration of fuel blends is another parameter that affects the cylinder pressure. In this regard, the application biofuels leads to earlier combustion and improve the cylinder pressure due to increasing the oxygen content of the fuel blends^35^. According to Fig. 3, the kinematic viscosity of fuel blends increases by increasing the WFO biodiesel content and decreasing the bioethanol content of fuel blends, respectively. As an advantage, the viscosity of waste fish oil biodiesel is lower compared to calophyllum biodiesel by 87% and pongamia biodiesel by 48%^8^. It should be also noted that excessive reduction in the viscosity of fuel mixture is not desirable because it can be leads to leakage in the fuel system^36^. Therefore, the viscosity of fuel blends can be adjusted to the desired value within the standard range by adjusting the bioethanol and WFO biodiesel contents of the ternary fuel blends. It was also found that the B5E15 blends has the lowest value of kinematic viscosity.

Figures 4 and 5 show the pour point and cloud point of different fuel samples, respectively. Pour point and cloud point are important properties for using the fuel samples in cold climate conditions. Lack of attention to these parameters can leads to the crystal formation and subsequent blockage of the fuel lines at low temperatures^37^. As can be seen, an increment in the WFO biodiesel content of ternary fuel blends results in an increase in the pour point and cloud point of the fuel blends. Pour point and cloud point of WFO biodiesel is higher compared to petro-diesel, which is inappropriate for the engine performance in the cold weather conditions^38^. Pour point and cloud point of WFO biodiesel is more suitable for preparation of the fuel blends compared to other biodiesel samples. For example, the pour point and the cloud point of palm biodiesel are 15 °C and 16 °C respectively which are obviously higher compared to WFO biodiesel pour point and cloud point^8^. On the other hand, an increment in the bioethanol content of ternary fuel blends results in a decrease in the pour point and cloud point of fuel blends. Therefore, the cold properties of fuel blends can be tuned within the desired range by adjusting the WFO biodiesel and bioethanol content of the fuel blend. In this regard, the presence of bioethanol in the fuel blends can reduce the incremental impact of WFO biodiesel on the pour point and cloud point of ternary fuel blends to some extent^38^.

Figure 6 shows the flash point of different fuel samples. The flash point is an important property from safety point of view related to handling, storage, and transportation of fuel blends^39^. According to Fig. 6, an increment in the WFO biodiesel content of ternary fuel sample results in a slight increment in the flash point of the ternary fuel blends. Residual alcohol content, number of carbon atoms, and double bonds of the biodiesel are the most important factors that influence the biodiesel flash point^40^. However, increasing the bioethanol content of the fuel blends results in a considerable decrease in the flash point of the fuel blend. Generally, the blend component with the lowest flash point has the dominant effect on the fuel blend flash point. It should be noted that the addition of bioethanol in 0–10% range results in a noticeable reduction in the boiling point range of the fuel blend^19,41^. As can be observed, the flash point of fuel blends is mainly influenced by the bioethanol content. The average value of flash point for different fuel blends is about 34 °C, which is lower that petro-diesel and WFO biodiesel. Therefore, special cares should be applied in the transportation and storage of the fuel blends containing bioethanol.

Figure 7 shows the heat value of different fuel samples. As can be observed, an increase in the WFO biodiesel and bioethanol contents of the ternary fuel blend results in a decrease in the heat value of fuel blends. This finding can be explained by the lower heat value of WFO biodiesel and bioethanol compared to petro-diesel. The carbon chain length affects the heat value of fuel samples. In this regard, the longer carbon chain length results in the higher heat value of the fuel sample^42^. Therefore, addition of WFO biodiesel and bioethanol to the ternary fuel blends results in a decrement in the heat value of the fuel blend. In this regard, B5E5 has the highest heat value considering different examined fuel blends. Besides, it was found that the heat value of the ternary fuel blends is decreased by 7% in average compared to the neat petro-diesel.

**Figure 8:**
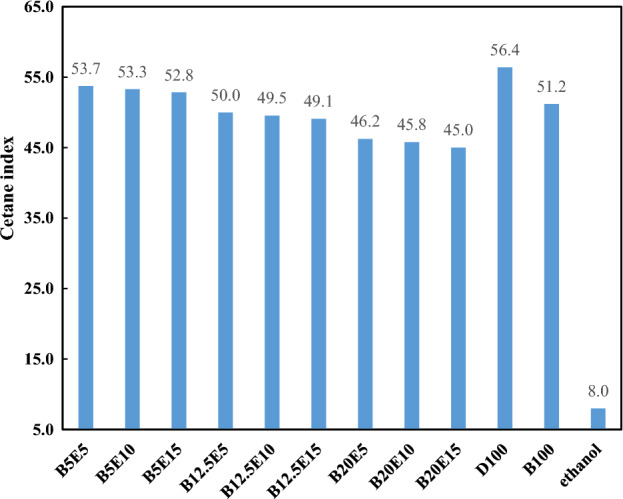
Cetane index of different ternary fuel blends.

Figure 8 shows the Cetane index of different examined fuel samples. As can be observed in Fig. 8, the addition of WFO biodiesel and bioethanol to the fuel blend leads to a subsequent decrease in the Cetane index. It should be noted that addition of bioethanol to the fuel blends leads to a considerable decrement in the Cetane index of fuel blends due to its too low Cetane index. Bizzo and Moretti^43^ also reported Cetane number decrement as a result of increment of the ethanol content of fuel blend. It should be noted that an increment in the Cetane index of fuel blend results in a decrease in the ignition delay and more rapid combustion during the premixed combustion phase, which results in consequent higher cylinder pressure. Therefore, in cylinder pressure of fuel blends is decreased by increasing the bioethanol and WFO biodiesel contents of fuel blends^31,44,45^.

### Engine performance

After evaluation of different physicochemical specifications of the fuel blends, the engine performance parameters including power, torque, brake thermal efficiency, and engine specific fuel consumption were investigated in a single-cylinder diesel engine at the speed of 1800 rpm and full load condition.

#### Brake power and torque

**Figure 9:**
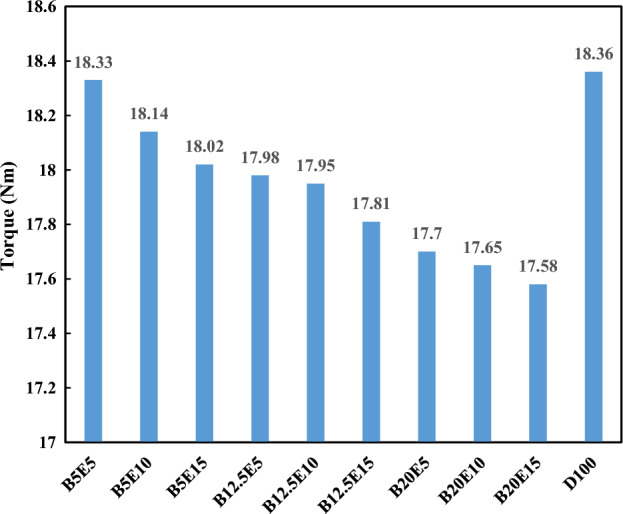
Engine torque using different ternary fuel blends.

**Figure 10:**
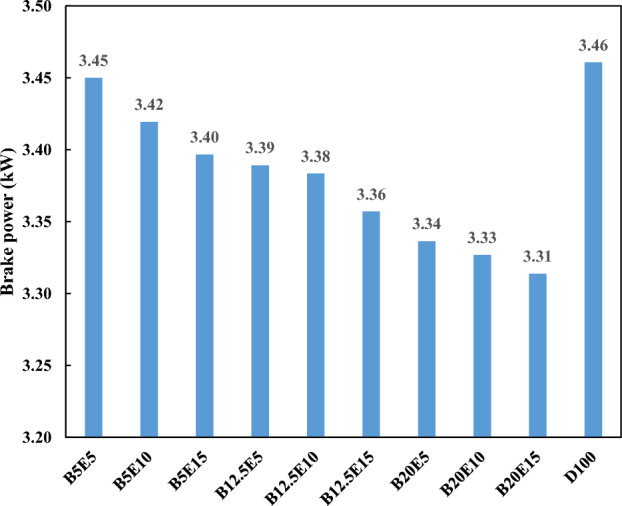
Break power using different ternary fuel blends.

Figures 9 and 10 show the engine torque and brake power using different ternary fuel blends containing different percentages of diesel, WFO biodiesel, and bioethanol, respectively.

As mentioned in the previous section, the heat value of WFO biodiesel is lower compared to petro-diesel. The heat value of bioethanol is also lower compared to WFO biodiesel. Therefore, an increment in the WFO biodiesel and bioethanol contents of the ternary fuel blends leads to a decrease in the engine torque and brake power. The lower heat value of the fuel blends containing WFO biodiesel and bioethanol leads to lower energy output in the combustion process and subsequent lower brake power and torque^4,46^. The application of fuel blends containing different concentration of bioethanol at constant concentration of 5%, 12.5%, and 20% of WFO biodiesel results in a reduction in the engine torque by 1.1%, 2.4%, and 3.9%, respectively. For these fuel blends, the engine break power also reduces by 0.8%, 2.3%, and 3.7%, respectively. Using the fuel blends containing different concentration of WFO biodiesel at constant concentration of 5%, 10%, and 15% of bioethanol results in a decrement in the engine torque by 1.9%, 2.4%, and 3% and break power by 1.7%, 2.3%, and 2.9%, respectively.

#### Brake specific fuel consumption (BSFC)

**Figure 11:**
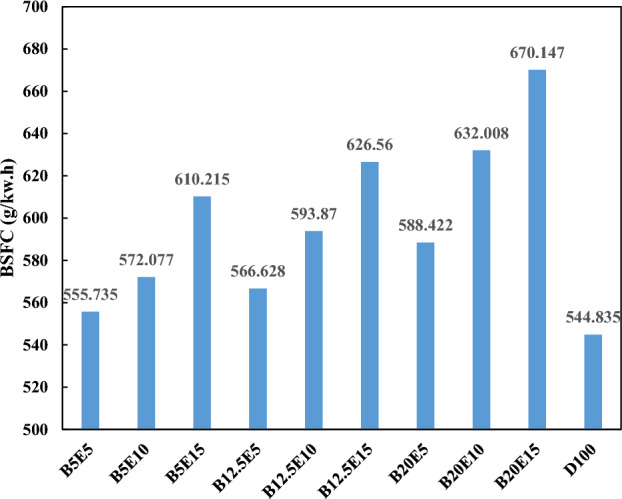
BSFC using different ternary fuel blends.

Figure 11 shows BSFC of different examined ternary fuel blends. Break specific fuel consumption is the ratio of the fuel consumption rate to the generated power. BSFC, as a measure for the evaluation of the fuel efficiency, depends on the heat value, density, and viscosity of fuel^4,46,47^. As can be seen in Fig. 11, an increment in the WFO biodiesel content of the ternary fuel blends result in a subsequent increase in the BSFC. This can be attributed to the decrement in the efficiency due to lower heat value, higher density, and higher viscosity of WFO biodiesel compared to the petro-diesel. An increment in the bioethanol content of the ternary fuel blends also results in an increase in BSFC. Bioethanol has lower density and viscosity compared to WFO biodiesel and petro-diesel. Therefore, an enhancement in the fuel and air mixing is expected by addition of bioethanol to the fuel blends. However, the considerable lower heat value of bioethanol compared to WFO biodiesel and petro-diesel results in a subsequent increase in the BSFC^12,48,49^. Fang et al.^50^, Zhu et al.^51^, and Al-Hassan et al.^52^ reported an increase in the BSFC by an increase in the ethanol content of fuel blends. It should be mentioned that WFO biodiesel and bioethanol addition to the ternary fuel blends leads to about 10% on average increment in the BFSC compared to petro-diesel.

#### Brake thermal efficiency (BTE)

**Figure 12:**
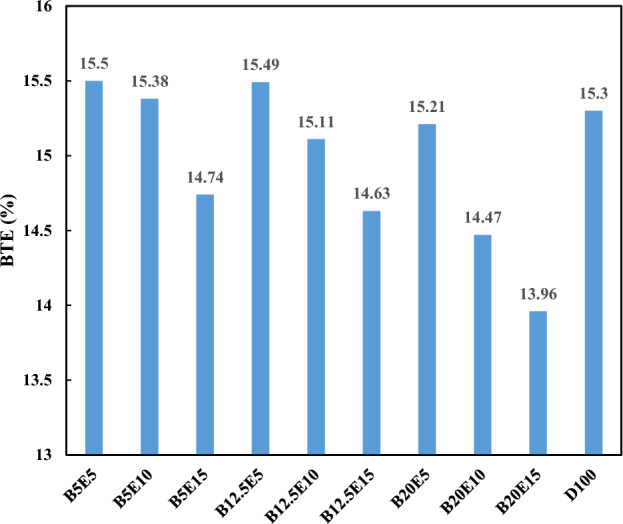
BTE using different ternary fuel blends.

Figure 12 shows BTE for different examined fuel blends. BTE is a parameter that represent the efficiency of the fuel energy conversion to the mechanical energy output^12^. BTE is influenced by BSFC and heat value of fuel, inversely. As can be observed in Fig. 12, the trend of BTE change for different fuel blends is opposite to the trend of BFSC. It was also found that BTE for B5E5, B5E10, and B12.5E5 fuel blends is higher compared to the neat petro-diesel in spite of their higher BSCF compared to petro-diesel. This can be explained by the lower heat value of these ternary fuel blends in comparison with the petro-diesel, which dominate the effect of BFSC on BTE. The results also show that an increment in the bioethanol content of the ternary fuel blends results in a decrease in BTE.

### Engine exhaust emission

#### CO emission

**Figure 13:**
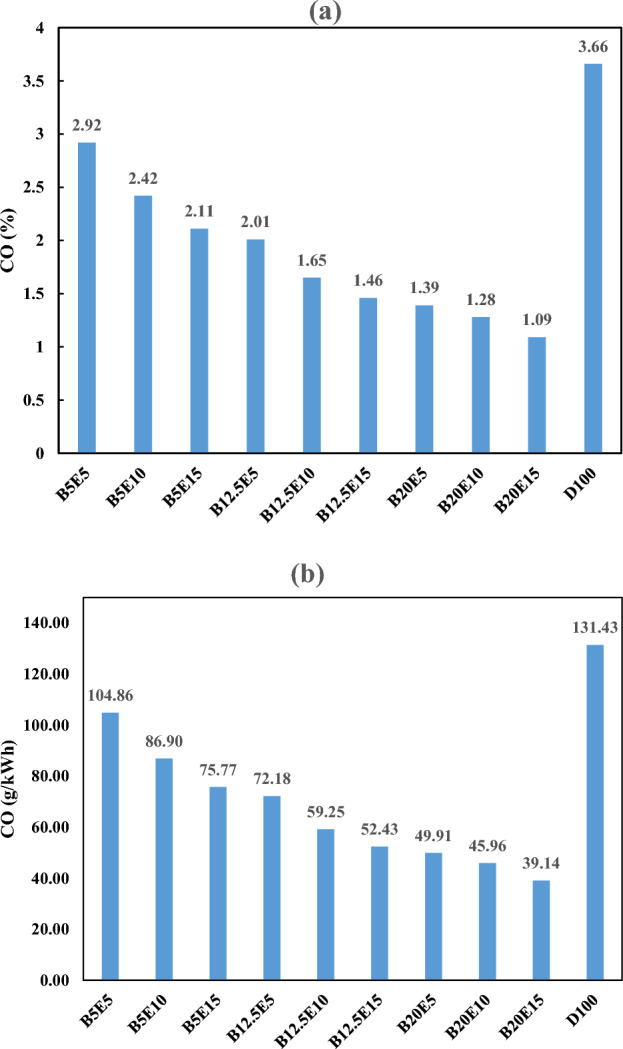
CO emission using different ternary fuel blends (**a**) % (**b**) g/(kW h).

Figure 13 shows the CO emission in the engine exhaust for different examined ternary fuel samples. Figure 13a shows CO emission in %. Figure 13b shows CO emission in g/kWh, which is calculated by Eq. (6)^53^:

6 $$ {\text{CO}} \left( {\frac{{\text{g}}}{{{\text{kWh}}}}} \right) = 3.591*10^{ - 3} *{\text{CO}}\left( {{\text{ppm}}} \right) $$

The emission of CO, as a toxic gas, can be attributed to the incomplete combustion as a result of poor fuel and oxygen mixing. As can be observed in Fig. 13, the CO emission is decreased by an increment in the WFO biodiesel and bioethanol contents of the ternary fuel blends. This can be explained by an increment in the oxygen content of the fuel blends by increasing the WFO biodiesel and bioethanol content of the ternary fuel blends. Enrichment in the oxygen content of the ternary fuel blends results in more complete combustion due to further oxidation of CO in the combustion process. This finding is in agreement with Kwanchareon et al.^19^, Subbaiah et al.^2^, Li et al.^54^, Shi et al.^55^, and Guarieiro et al.^10^ studies regarding the CO emission in full load engine performance. According to Fan et al.^50^ and Zhu et al.^51^ studies, the CO emission is increased using rape seed oil biodiesel/ethanol/diesel and waste cooking oil biodiesel/ethanol/diesel compared to the neat diesel fuel. However, a noticeable reduction in the CO emission is observed in the present study as an advantage of using WFO biodiesel in the ternary fuel samples. It should be noted that the CO emission of B5E5 (i.e., the fuel blends with the lowest WFO biodiesel and bioethanol content) is 20.22% lower compared to the petro-diesel. Besides, the CO emission of B20E15 (i.e., the fuel blends with the highest WFO biodiesel and bioethanol content) is 70.22% lower compared to the petro-diesel. This is a noticeable reduction in CO emission.

#### *CO*_*2*_* emission*

**Figure 14:**
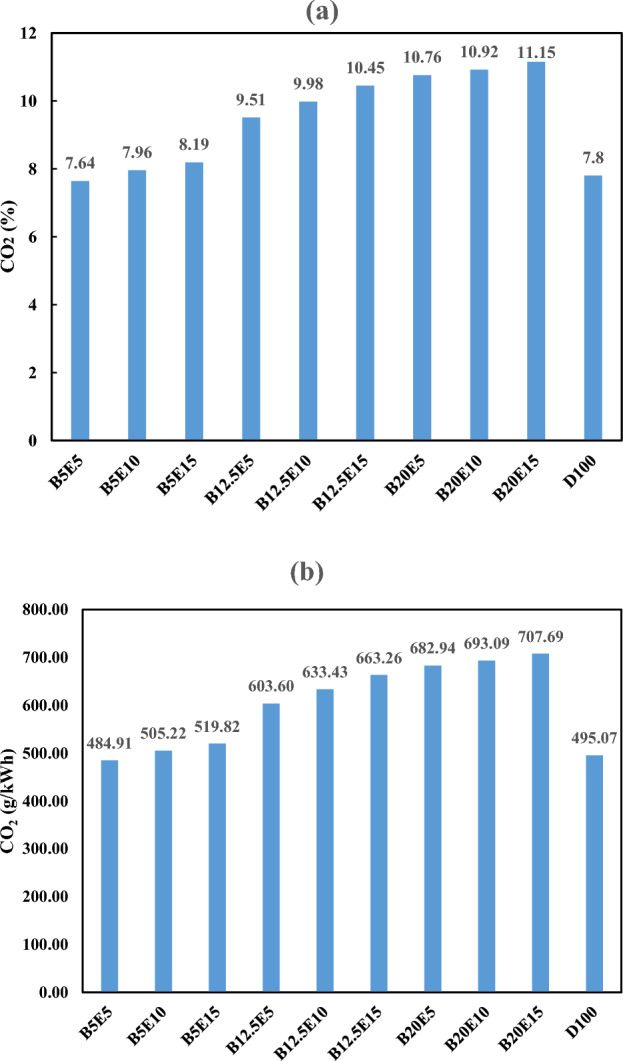
CO_2_ emission using different ternary fuel blends (**a**) % (**b**) g/(kW h).

Figure 14 shows the CO_2_ emission in the engine exhaust for different examined ternary fuel samples. Figure 14a shows CO_2_ emission in volume percent. Figure 14b shows CO_2_ emission in g/kWh which is calculated by Eq. (7)^53^.

7 $$ {\text{CO}}_{2} \left( {\frac{{\text{g}}}{{{\text{kWh}}}}} \right) = 63.470*{\text{CO}}_{2} \left( {{\text{vol}}\% } \right) $$

According to Fig. 14, CO_2_ emission in the engine exhaust is increased by increment in the WFO biodiesel and bioethanol content of the ternary fuel blend. As mentioned in the previous section, an increment in the oxygen content of the fuel blends as a result of WFO biodiesel and bioethanol addition leads to an enhancement in the complete combustion and subsequent increment in the CO_2_ emissions. This finding is in agreement with Subbaiah et al.^2^, Hulwan et al.^12^, Cheenkachorn et al.^56^, and Guarieiro et al.^10^ studies.

#### Unburned hydrocarbons (UHC) emission

**Figure 15:**
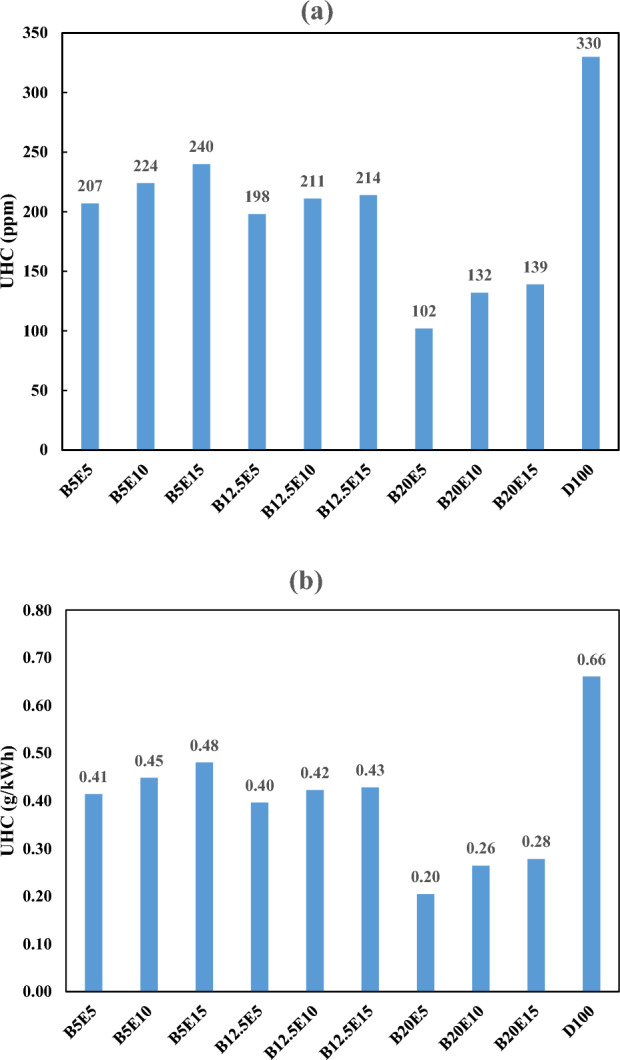
Unburned hydrocarbon emission using different ternary fuel blends (**a**) ppm (**b**) g/(kW h).

Figure 15 shows the UHC emission in the engine exhaust for different examined ternary fuel samples. Figure 15a shows UHC emission in ppm. Figure 15b shows UHC emission in g/kWh which is calculated by Eq. (8)^25^.

8 $$ {\text{HC}}\left( {\frac{{\text{g}}}{{{\text{kWh}}}}} \right) = 2.002*10^{ - 3} *{\text{HC}}\left( {{\text{ppm}}} \right) $$

As can be observed in Fig. 15, the UHC emission is significantly lower for all examined ternary fuel blends in comparison with the petro-diesel. An increase in the WFO biodiesel content of the fuel blends results in more complete combustion and subsequent lower UHC emission. According to the previous studies, the UHC emission is also decreased obviously by an increment in the biodiesel content of the fuel blend samples^19,57^. It was also found that increasing the bioethanol content of the fuel blends results in an increment in the UHC emission. This can be attributed the decrement in the combustion efficiency as a result of bioethanol increment in the fuel blends. The trend of BSFC with bioethanol content of the fuel blends also confirms this fact. According to Kwanchareon et al.^19^ and Subbaiah et al.^2^, this can be attributed to unburned bioethanol emission in the engine exhaust as a result of large ethanol dispersion region in the combustion chamber. According to Fang et al.^50^, the application of rape seed oil biodiesel in a biodiesel/alcohol/diesel blend results in an increment in UHC emission. However, as a clear advantage, the application of waste fish oil biodiesel in ternary fuel samples has superior performance compared to rape seed oil biodiesel.

#### *Nitrogen oxide (NO*_*x*_*) emission*

**Figure 16:**
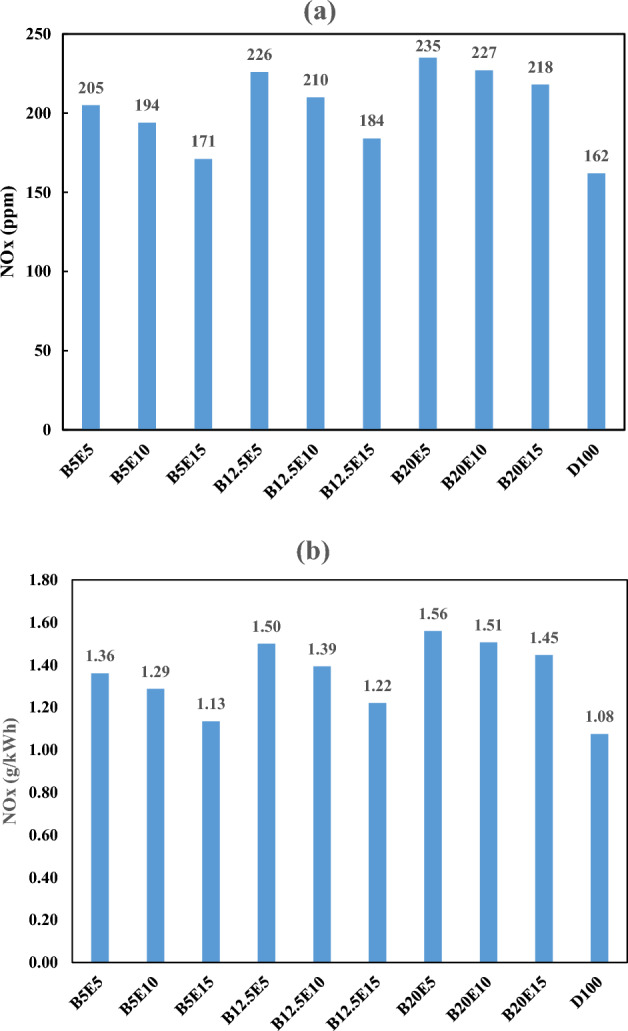
NO_x_ emission using different ternary fuel blends (**a**) ppm (**b**) g/(kW h).

Figure 16 shows the NO_x_ emission in the engine exhaust for different examined ternary fuel samples. Figure 16a shows NO_x_ emission in ppm. Figure 16b shows NO_x_ emission in g/kWh which is calculated by Eq. (9)^25^.

9 $$ {\text{NO}}_{x} \left( {\frac{{\text{g}}}{{{\text{kWh}}}}} \right) = 6.636*10^{ - 3} *{\text{NO}}_{x} \left( {{\text{ppm}}} \right) $$

As can be observed in Fig. 16, the NO_x_ emission is increased by an increment in the WFO biodiesel content of the ternary fuel blends. It is also found that an increment in the bioethanol content of the ternary fuel blends results in a decrement in the NO_x_ emission. The dominant mechanism of NO_x_ formation is thermal mechanism. Thermal mechanism includes the endothermic reactions, which stimulated by high temperature. Equation (10) gives NO formation rate based on Zeldovich mechanism which describes the NO formation by thermal mechanism^58^:

10 $$ \frac{{d\left[ {{\text{NO}}} \right]}}{dt} = \frac{{6.0 \times 10^{16} }}{{T^{0.5} }}{\text{exp}}\left\{ {\frac{ - 69090}{T}} \right\}\left[ {{\text{N}}_{2} } \right]\left[ {{\text{O}}_{2} } \right]^{0.5} $$

According to Eq. (10), the temperature and oxygen concentration are effective parameters in NO formation. In this regard, an increase in the temperature leads to an increment in NO formation. Increasing the oxygen concentration also leads to increasing NO formation because the oxygen as a reactant for NO_x_ formation reaction, is an essential agent for the endothermic reaction progress^3,58^.

Increasing biodiesel content in the fuel blends leads to the increment in the fuel blend oxygen content. The normal boiling point of biodiesel is about 350 °C and it ignites before evaporation, which leads to increasing the combustion chamber temperature. Therefore, the increment in the fuel blend oxygen content and combustion chamber temperature due to WFO biodiesel addition to the ternary fuel blends results in an increment in the NO_x_ emission^59^. Increasing bioethanol in the fuel blends also leads to the fuel blend oxygen content increment. However, the normal boiling point of bioethanol is about 78 °C and its latent heat of evaporation is 846 kJ/kg. It seems that a part of bioethanol content of the fuel blends evaporates during ignition in the combustion chamber, which leads to a noticeable reduction in the combustion chamber temperature. Therefore, the increment in the fuel blend oxygen content and the decrement in the combustion chamber temperature due to bioethanol addition to the fuel blends affect the NO formation rate oppositely. The decreasing trend of NO_x_ formation by increasing the bioethanol content of the fuel blends confirmed that the combustion chamber temperature decrement effect on the NO formation is dominant and therefore, the increment in the bioethanol content of the ternary fuel blends results in a decrement in the NO_x_ emission^7,12^.

This is an interesting pull–push effect, which can be applied to adjust the engine NO_x_ emission to the desired limit by adjusting the WFO biodiesel and bioethanol contents of the ternary fuel blends. In this regard, as can be observed, the NO_x_ emission of B5E15 fuel blend is in the range of the neat petro-diesel, approximately.

### Recommended ternary fuel formulation

According to Euro 5 diesel standard, B5E5, B5E10, and B5E15 fuel blends are the fuel samples which pass the standard criteria. These fuel sample can be considered as the potential candidates for the best fuel blends. B5E5 fuel blend has better performance compared to the other fuel blends considering the engine performance tests. B5E15 fuel blend has better performance compared to the other fuel blends considering the engine exhaust emissions.

**Figure 17:**
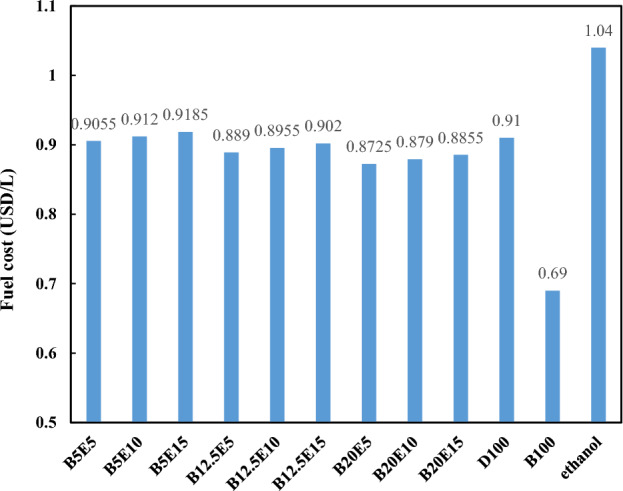
Preliminary cost evaluation for different ternary fuel blends.

According to Kara et al.^60^, the petro-diesel, and WFO biodiesel costs are 0.91 USD/L, 0.69 USD/L, respectively. According to IRENA reports^61^, the bioethanol cost is 1.04 USD/L. Therefore, a preliminary cost evaluation can be carried out for different ternary fuel blends. Figure 17 shows the outcome of this preliminary cost evaluation.

According to Fig. 17, cost of all fuel blends is comparable to the neat petro-diesel. It should be noted that the cost of WFO biodiesel, petrodiesel, and bioethanol is varied throughout the world as a results of balance between supply and demand of these fuel, the availability of raw materials and the employed production process. Therefore, more detailed cost analysis in different situations is highly recommended.

## Conclusion

In the presents study, the physicochemical specifications, engine performance, and engine exhaust were investigated for different ternary fuel blends containing WFO biodiesel, bioethanol, and petro-diesel. The main findings are as follows:

- It was found that the viscosity, density, cloud point, and pour point of all examined fuel blends are in standard range and close to the petro-diesel. However, the flash point of the fuel blends containing high bioethanol content is considerably lower compared to the neat petro-diesel due to low flash point of bioethanol. This should be considered in the storage and handling of the fuel blends. The calorific value of the fuel blends containing WFO biodiesel and bioethanol is lower compared to the neat petro-diesel.
- Regarding the engine performance parameters, it was found that the engine torque and brake power is lower for the ternary fuel blends containing WFO biodiesel and bioethanol compared to the neat petro-diesel. The BSFC was higher for the fuel blends compared to the neat petro-diesel is used. This can be attributed to the lower heat value of the ternary fuel blends compared to the neat petro-diesel.
- Regarding the engine exhaust emission, a significant decrement in the CO emission and UHC emission was observed for the ternary fuel blends compared to the neat petro-diesel. This is a clear advantage of using WFO biodiesel/bioethanol/diesel fuel blends compared to the neat petro-diesel. It was also found that despite the increment in NO_x_ emission using the fuel blends containing WFO biodiesel and bioethanol, the engine NO_x_ emission could be adjusted to the desired limit by tuning the WFO biodiesel and bioethanol content of the ternary fuel blends.
- B5E5 fuel blend has the best performance compared to the other fuel blends considering the engine performance parameters. Moreover, B5E15 is the best fuel blend considering the engine exhaust emissions. Investigation of the influence of the engine rpm and load on the performance and emission of different fuel blends is an interesting subject for future studies.

### Supplementary Information

Supplementary Information.

## Data Availability

It should be justified that “All data generated or analysed during this study are included in this published article [and its supplementary information files]”.
