# Supplementary material for: Physical properties, engine performance, and exhaust emissions of waste fish oil biodiesel/bioethanol/diesel fuel blends
Source: Sci Rep. 2023 Aug 28;13:14024. doi: 10.1038/s41598-023-41280-5 (PMC10462698; doi:10.1038/s41598-023-41280-5)
Supplement: Supplementary file 1 — Supplementary Information. [file 41598_2023_41280_MOESM1_ESM.docx]

**Supporting Information**

**For**

Physical properties, Engine performance, and Exhaust emissions of Waste fish oil biodiesel/bioethanol/diesel fuel blends

**Table I. Technical specifications of the employed diesel engine**

| **Model** | 3LD510 |
| --- | --- |
| **manufacturing factory** | Lombardini Italy company |
| **Number of cylinder** | 1 |
| **Cylinder course** | 90 mm |
| **Cylinder diameter** | 85 mm |
| **Cylinder volume** | 510 cm^3^ |
| **Maximum power at 3000 rpm** | 12.2 Hp ( 9 kW) |
| **Maximum torque at 1800 rpm** | 33 N.m |
| **compression ratio** | 1 : 17.5 |

**Table II: The specifications of dynamometer**

| **Power magnitude accuracy** | ± 0.1 kW |
| --- | --- |
| **torque magnitude accuracy** | ± 0.1 N.m |
| **Rotational speed magnitude accuracy** | ± 0.1 rpm |
| **Maximum power measurement** | 21 hp |
| **Maximum torque measurement** | 80 N.m |
| **Maximum speed measurement** | 10000 rpm |

**Table III. The specifications of emission sensors**

| Measurable gases | CO | CO_2_ | HC | O_2_ | NO_X_ |
| --- | --- | --- | --- | --- | --- |
| Measuring range | 0-15%  % volume | 0-20%  % volume | 0-2000  ppm volume | 0-25%  % volume | 0-5000  ppm volume |
| Measurement accuracy | 0.03%  % volume | 0.5%  % volume | 10  ppm volume | 0.1%  % volume | 32-120  ppm volume |
| Measurement method | Infrared | Infrared | Infrared | Electronic | Electronic |

**Table IV. Different fatty acid esters in the waste fish oil biodiesel**

| **Composition** | **% wt** |
| --- | --- |
| C14 | 8.06 |
| C15 | 2.09 |
| C16 | 28.14 |
| C16:1 | 8.41 |
| C17 | 2.37 |
| C17:1 | 1.89 |
| C18 | 5.02 |
| C18:1 | 18.26 |
| C19 | 4.9 |
| EPA | 6.32 |
| DHA | 10.3 |
| Others | 4.14 |
